# Supplementary material for: Effect of Three Bakery Products Formulated with High-Amylose Wheat Flour on Post-Prandial Glycaemia in Healthy Volunteers
Source: Foods. 2023 Jan 9;12(2):319. doi: 10.3390/foods12020319 (PMC9857412; doi:10.3390/foods12020319)
Supplement: Supplementary file 1 [file foods-12-00319-s001.zip › foods-2141656-supplementary.pdf]

Consumer Test Questionnaire

Full survey (translated from Italian)

Thank you for agreeing to participate in this survey. The survey has no commercial or promotional purposes. The questionnaire is anonymous, the information respects the Privacy Act (Decree No. 101/2018) and the data collected will not be used for commercial or promotional purposes.

*Required field

1. ENTER HERE THE CODE PROVIDED UPON ACCEPTANCE *

   ____

2. Indicate on a scale of 1 to 9 how hungry you feel*

I am not hungry at all								I am very hungry
1	2	3	4	5	6	7	8	9

PLEASE, OPEN THE PACKAGE WITH THE LETTER A

LOOK at the product and indicate how appropriate you find the following statements to express the characteristics of the product (from "not at all" to "completely"), using the scale below

	Not at all								Completely
3. It is yellow*	1	2	3	4	5	6	7	8	9

	Not at all								Completely
4. It is brown*	1	2	3	4	5	6	7	8	9

	Not at all								Completely
5. It is large*	1	2	3	4	5	6	7	8	9

	Not at all								Completely
6. It is small*	1	2	3	4	5	6	7	8	9

	Not at all								Completely
7. It is thick*	1	2	3	4	5	6	7	8	9

	Not at all								Completely
8. It is thin*	1	2	3	4	5	6	7	8	9

TASTE the product and indicate how appropriate you find the following statements to express the characteristics of the product (from "not at all" to "completely"), using the scale below
taste at least one biscuit before answering

	Not at all								Completely
9. It is crumbly*	1	2	3	4	5	6	7	8	9
10. It is chewy*	1	2	3	4	5	6	7	8	9
11. It is crispy*	1	2	3	4	5	6	7	8	9
12. It is doughy*	1	2	3	4	5	6	7	8	9
13. It is dry*	1	2	3	4	5	6	7	8	9
14. It is stringy *	1	2	3	4	5	6	7	8	9
15. It is fatty *	1	2	3	4	5	6	7	8	9
16. It is salty *	1	2	3	4	5	6	7	8	9
17. It is sweet *	1	2	3	4	5	6	7	8	9
18. It is fragrant *	1	2	3	4	5	6	7	8	9
19. It is toasted *	1	2	3	4	5	6	7	8	9
20. It is bitter *	1	2	3	4	5	6	7	8	9

21. Rate your overall liking of the product from dislike extremely (1) to like extremely (9)*

dislike extremely								like extremely
1	2	3	4	5	6	7	8	9

22. How much would you be willing to pay for a snack pack of the product you have just tasted? Please write the amount in € *____

NOW DRINK SOME WATER TO CLEAN YOUR MOUTH AND THEN OPEN THE PACKET WITH THE LETTER B

LOOK at the product and indicate to what extent you find the following statements appropriate to express the characteristics of the product (from 'not at all' to 'completely'), using the reference scale below

	Not at all								Completely
23. It is yellow*	1	2	3	4	5	6	7	8	9

	Not at all								Completely
24. It is brown*	1	2	3	4	5	6	7	8	9

	Not at all								Completely
25. It is large*	1	2	3	4	5	6	7	8	9

	Not at all								Completely
26. It is small*	1	2	3	4	5	6	7	8	9

	Not at all								Completely
27. It is thick*	1	2	3	4	5	6	7	8	9

	Not at all								Completely
28. It is thin*	1	2	3	4	5	6	7	8	9

TASTE the product and indicate how appropriate you find the following statements to express the characteristics of the product (from "not at all" to "completely"), using the scale below
taste at least one biscuit before answering

	Not at all								Completely
29. It is crumbly*	1	2	3	4	5	6	7	8	9
	Not at all								Completely
30. It is chewy*	1	2	3	4	5	6	7	8	9
	Not at all								Completely
31. It is crispy*	1	2	3	4	5	6	7	8	9
	Not at all								Completely
32. It is doughy*	1	2	3	4	5	6	7	8	9
	Not at all								Completely
33. It is dry*	1	2	3	4	5	6	7	8	9
	Not at all								Completely
34. It is stringy *	1	2	3	4	5	6	7	8	9
	Not at all								Completely
35. It is fatty *	1	2	3	4	5	6	7	8	9
	Not at all								Completely
36. It is salty *	1	2	3	4	5	6	7	8	9
	Not at all								Completely
37. It is sweet *	1	2	3	4	5	6	7	8	9
	Not at all								Completely
38. It is fragrant *	1	2	3	4	5	6	7	8	9
	Not at all								Completely
39. It is toasted *	1	2	3	4	5	6	7	8	9
	Not at all								Completely
40. It is bitter *	1	2	3	4	5	6	7	8	9

41. Rate your overall liking of the product from dislike extremely (1) to like extremely (9) *

dislike extremely								like extremely
1	2	3	4	5	6	7	8	9

42. How much would you be willing to pay for a snack pack of the product you have just tasted? Please write the amount in €* ____

43. Overall, which of the two products you tasted did you like best? *

- o Product A
- o Product B

ONE OF THE PRODUCTS YOU TASTED CONTAINS NATURAL HIGH AMYLOSE FLOURS, WHICH RAISE BLOOD SUGAR LESS THAN A NORMAL PRODUCT.

44. How much would you be willing to pay for a packet-snack of product containing natural high amylose flours? Please write the amount in € * ______________________________

THE PRODUCT WITH HIGH AMYLOSE NATURAL FLOURS THAT YOU TASTED WAS THE ONE IN PACK B *[or A]*.

45. How much would you be willing to pay for a packet-snack of the B-package *[or A-package]* product now that you know it has beneficial properties for your health? Please write the amount in € *____

46. If you found this type of product on the market, how likely would you be to buy it? *
    Mark only one answer
    - o Definitely yes
    - o Probably yes
    - o Probably no
    - o Definitely no
    - o I don’t know

47. Would you recommend the of the product with high amylose flours? *
    Mark only one answer
    - o Definitely yes
    - o Probably yes
    - o Probably no
    - o Definitely no
    - o I don’t know
